# When Pain Relief Turns Perilous: Uncovering the Hidden Dangers and Advanced Management of Benzocaine-Induced Methemoglobinemia

**DOI:** 10.7759/cureus.67949

**Published:** 2024-08-27

**Authors:** Anam Umar, Muhammad Bilal, Bilal Jawed, Amber E Faquih

**Affiliations:** 1 Internal Medicine, Ascension St. Vincent's Birmingham, Birmingham, USA; 2 Internal Medicine, Jinnah Postgraduate Medical Centre, Karachi, PAK; 3 Infectious Diseases, University of Alabama at Birmingham, Birmingham, USA

**Keywords:** medical icu, methylene blue infusion, local anaesthetic, pulmonary critical care, acute hypoxemic respiratory failure, acquired methemoglobinemia, benzocaine

## Abstract

Benzocaine-induced methemoglobinemia is a rare complication associated with benzocaine, a local anesthetic known for its rapid pain relief. Acquired methemoglobinemia occurs when hemoglobin is oxidized to a ferric state, impairing oxygen binding. This condition can lead to respiratory distress and potentially fatal outcomes if not promptly diagnosed and treated.

We present the case of a 61-year-old Caucasian female admitted with respiratory distress after lumbar stenosis surgery. She developed acute hypoxemic respiratory failure due to pneumonia. Although initially responsive to antibiotics and oxygen, her condition worsened overnight despite non-invasive bilevel-positive airway pressure (BiPAP) therapy. The use of benzocaine spray for throat pain led to suspected methemoglobinemia. She was treated with high-dose vitamin C and methylene blue, resulting in significant improvement. This case report aims to raise awareness among healthcare workers and emphasizes that timely recognition and management are crucial for better outcomes.

## Introduction

Benzocaine is a widely used local anesthetic that provides effective topical pain relief in various medical settings. It works by temporarily blocking sodium channels in neuronal membranes, preventing cellular depolarization, slowing signal transmission, and reducing the likelihood of action potentials. Benzocaine typically begins to take effect within 30 seconds and is rapidly metabolized, with its primary excretion occurring through the urine [1]. It is available in several forms and concentrations, including solutions, tablets, sprays, and gels, commonly in strengths of 5%, 10%, and 20%. Its over-the-counter availability and efficacy make it popular for alleviating discomfort from endoscopies, dental work, and minor skin irritations [2]. However, benzocaine use carries risks, including the potential for methemoglobinemia, a rare but serious condition that impairs the body’s ability to deliver oxygen. Prompt recognition and intervention are crucial, especially in critical care settings where patients may be more susceptible [3].

## Case presentation

**Figure 1:**
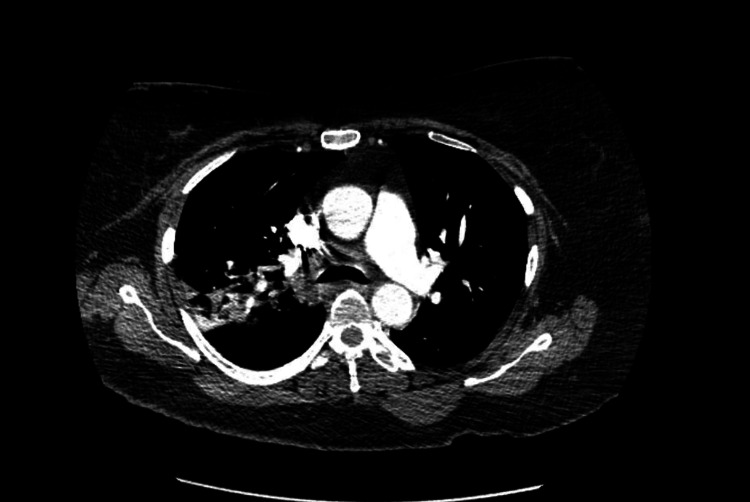
CTA chest The findings of the CTA chest are as follows: Pulmonary arteries are well opacified. There is no evidence of pulmonary thromboembolism. The thoracic aorta is well opacified. There is no evidence of aneurysm or dissection. No mediastinal or hilar adenopathy of significance. Heart size is borderline enlarged and there is no pericardial effusion. Also, there is minimal coronary artery calcification. There is significant airspace disease in the night upper lobe consistent with pneumonia. There is also pulmonary opacity in the right lower lobe also suspicious for pneumonia. The left lung is grossly clear other than some minimal atelectasis. No pleural effusion in either lung. Imaging obtained through the upper abdomen does not demonstrate any acute finding. There was no acute osseous abnormality. CTA: Computed tomography angiography

**Figure 2:**
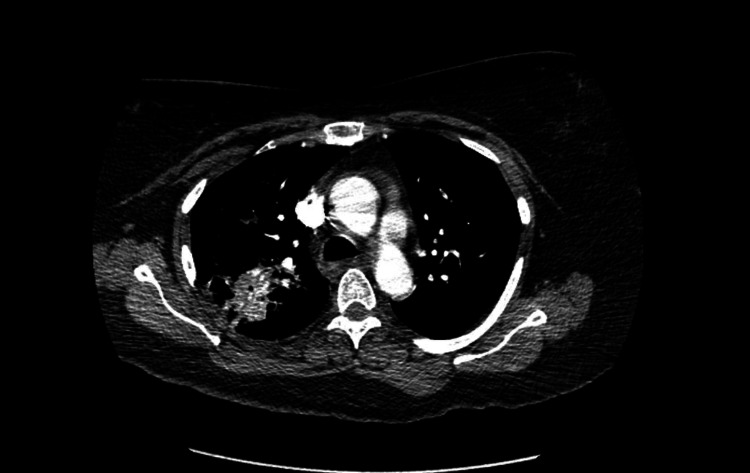
CTA of the chest The impression of the imaging is as follows: Large area of airspace disease within the right upper lobe most likely due to bacterial pneumonia Probable second area of pneumonia within the right lower lobe. The left lung is grossly clear other than some slight atelectasis. No evidence for PTE. CTA: Computed tomography angiography; PTE: pulmonary thromboendarterectomy

A 61-year-old Caucasian female with a complex medical history, including chronic obstructive pulmonary disease, asthma, anxiety disorder, and tobacco abuse, presented with respiratory distress following surgical intervention for severe lumbar stenosis at L4-5 and L5-S1, with associated foraminal stenosis. This led to her admission to the hospitalist service and a pulmonary consultation. Diagnostic evaluation revealed acute hypoxemic respiratory failure secondary to pneumonia (Figures 1, 2), necessitating the initiation of broad-spectrum antibiotics and supplemental oxygen.

Critical event

Despite some initial signs of improvement, the patient's condition took a sudden and alarming turn during her hospitalization. Overnight, her respiratory distress worsened dramatically, and her oxygen saturation levels plummeted into the dangerously low range of the 70s and 80s. Despite treatment with a non-invasive bilevel-positive airway pressure (BiPAP), oxygenation did not improve. Upon reviewing the events preceding this rapid deterioration, it was discovered that the patient had received a dose of benzocaine spray for throat pain. Given benzocaine's association with rare methemoglobinemia, suspicion arose that benzocaine-induced methemoglobinemia might have caused the unexpected and concerning decline in oxygen saturation levels, The arterial blood gas (ABG) results revealed a methemoglobin level of 42.8% (normal 0-1.5%), while oxyhemoglobin was 56.1 % (normal 94-99%).

Treatment and response

In response to the suspected diagnosis of benzocaine-related methemoglobinemia, the medical team promptly initiated treatment with high-dose vitamin C and methylene blue. This intervention resulted in an improvement in the patient's symptoms, underscoring the critical importance of timely recognition and management of this rare but serious complication associated with benzocaine use.

## Discussion

Methemoglobinemia can manifest either congenitally or as an acquired condition. The congenital form arises from enzyme defects, genetic mutations affecting the hemoglobin protein, or metabolic irregularities (Table 1) [4].

**Table 1:** Congenital methemoglobinemia Congenital methemoglobinemia can be categorized based on its causes, types, and inheritance patterns. Hemoglobin M (Hb M) disease results from defects in the hemoglobin alpha or beta globin chains and follows an autosomal dominant inheritance pattern. In contrast, cytochrome b5 deficiency includes two types: cytochrome b5 reductase deficiency (RBC type) and type II cytochrome b5 reductase deficiency (generalized type). Both types of cytochrome b5 deficiency are caused by defects in the enzyme cytochrome b5 reductase (Cyb5R) and follow an autosomal recessive inheritance pattern. Table Credits: Amber Ehsan Faquih

Type of Congenital Methemoglobinemia	Cause	Types	Inheritance Pattern
Hemoglobin M (Hb M) disease	Defect in hemoglobin alpha or beta globin molecule	-	Autosomal dominant
Cytochrome b5 deficiency	Defect in Cytochrome b5: Enzyme/Protein Defect- Cytochrome b5 reductase (Cyb5R)	Type I Cytochrome b5 Reductase Deficiency (RBC type). Type II Cytochrome b5 Reductase Deficiency (Generalized type)	Autosomal recessive

Acquired methemoglobinemia, although rare, poses a significant risk associated with the use of local anesthetics. According to Joanne Guay's meta-analysis of 242 episodes, including nine cases involving repeated occurrences, benzocaine was identified as the cause in 66% of methemoglobinemia cases related to local anesthetics, while lidocaine was responsible for 5% [5]. Exposure to oxidizing agents such as dapsone, antimalarial drugs, and topical anesthetics like benzocaine, lidocaine, and prilocaine can lead to acquired methemoglobinemia. Additionally, nitrates and aniline dyes are known culprits [6]. In adults, the primary cause of acquired methemoglobinemia often stems from exposure to benzocaine spray during endoscopic procedures. Numerous case reports have highlighted benzocaine's prominence among local anesthetics, constituting approximately 66% of acquired methemoglobinemia cases attributed to this group of drugs [7]. Several risk factors contribute to the development of acquired methemoglobinemia from local anesthetics, including extremes of age (very young or old), presence of comorbidities, baseline hypoxia, and exceeding the dosage recommended by the manufacturer [8]. Recognizing and promptly treating patients with risk factors is crucial, especially when they present with cyanosis unresponsive to oxygen. A recent case report of a 60-year-old male with COVID-19 and multiple comorbidities highlights the critical importance of recognizing methemoglobinemia, which describes the patient’s deteriorating respiratory status and a significant "saturation gap," where pulse oximetry showed an oxygen saturation of 50% while arterial blood gas analysis indicated 100% saturation on positive pressure ventilation. This discrepancy suggested methemoglobinemia (as methemoglobin (MetHb) can cause pulse oximetry readings to stabilize around 85% due to its unique absorption properties). Despite initiating treatment with methylene blue based on clinical suspicion, a delay in diagnosis and administration rendered it ineffective, leading to patient's death. Methemoglobinemia was confirmed the next day of demise with a 9% MetHb level, with potential contributions from supplements like CV Support Formula containing ginkgo biloba, which can increase red blood cell fragility and trigger MetHb formation. This case underscores the urgency of timely diagnosis and treatment of methemoglobinemia, particularly when there is a noticeable saturation gap [9].

The emergence of symptoms associated with methemoglobinemia is closely tied to the level of methemoglobin detected in the bloodstream. Under normal conditions, methemoglobin levels typically fall within the range of 1% to 3%. Symptoms become noticeable once these levels surpass 10%, with levels exceeding 30% posing a significant threat to life [10]. Cyanosis in methemoglobinemia occurs due to an abnormal form of hemoglobin that is unable to carry oxygen. This leads to a reduced supply of oxygen to tissues, resulting in cyanosis and hypoxia. the cyanosis typically becomes apparent when methemoglobin levels exceed 10%. Within the range of 20% to 50% methemoglobin levels, patients may experience sensations like dizziness, headaches, fatigue, and respiratory distress. As methemoglobin levels approach 50%, patients may exhibit signs of lethargy and stupor, with mortality rates increasing significantly at levels exceeding 70% (Table 2) [11,12]. High levels of methemoglobin can lead to severe complications, including cardiovascular failure, coma, and seizures [13]. Methemoglobinemia should be suspected in patients presenting with cyanosis without cardiopulmonary causes and who are unresponsive to supplemental oxygen, as well as exhibiting previously discussed signs and symptoms. The presence of chocolate-colored blood is also indicative of methemoglobinemia. However, a definitive diagnosis is achieved through ABG analysis with co-oximetry. A co-oximeter employs multiple wavelengths to accurately measure the concentrations of oxyhemoglobin, carboxyhemoglobin, deoxyhemoglobin, and methemoglobin using spectrophotometric techniques [14]. 

**Table 2:** Signs and symptoms of methemoglobinemia at different levels Reprinted from [11]. Copyright (2014), with permission from Elsevier

Methemoglobin Concentration (g/dL)	Percentage of Total Hemoglobin (%)	Symptoms
< 1.5	< 10	None
1.5-3.0	10-20	Mild symptoms: Cyanotic (blue/slate gray) skin discoloration, chocolate brown blood, no hypoxic symptoms
3.0-4.5	20-30	Anxiety, light-headedness, headache, tachycardia, breathlessness, syncopal attacks
4.5-7.5	30-50	Fatigue, confusion, dizziness, tachypnea, increased tachycardia, weakness
7.5-10.5	50-70	Coma, seizures, arrhythmias, lactic acidosis, CNS depression

The initial treatment for methemoglobinemia involves discontinuing the offending medication, administering supplemental oxygen, and providing supportive care. Additional intervention is necessary if the patient exhibits severe symptoms, has a methemoglobin level greater than 30%, or has pulmonary and cardiac comorbidities. The preferred treatment is methylene blue, which acts as a reducing agent within red blood cells by converting ferric ion (Fe+++) back to its oxygen-carrying ferrous state (Fe++) [15]. The recommended dose of methylene blue for both children and adults is an intravenous infusion of 1-2 milligrams per kilogram of a 1% solution, administered over 5 to 30 minutes. The most common adverse effects of methylene blue include irritation with urination or pain at the intravenous site. When administered with serotonergic drugs, there is a risk of serotonin syndrome. Other potential central nervous system-related adverse effects in adults include dizziness, headache, coma, and confusion [16]. In neonates, methylene blue can affect the respiratory system and may also cause hemolytic jaundice [17]. Ascorbic acid, also known as vitamin C, is used as a second-line treatment when methylene blue is unavailable or in cases of hypersensitivity to methylene blue, severe renal impairment, pregnancy, or glucose-6-phosphate dehydrogenase (G6PD) deficiency. In patients with G6PD deficiency, methylene blue can induce hemolytic anemia, making ascorbic acid a safer alternative [4,15]. Ascorbic acid is a strong reducing agent that directly converts ferric ions (Fe+++) to ferrous ions (Fe++). It is administered intravenously at a dose of 1-10 grams every six hours until methemoglobin levels normalize. Unlike methylene blue, ascorbic acid does not have major side effects. In cases of methemoglobinemia that do not respond to methylene blue or ascorbic acid, treatments such as hyperbaric oxygen and exchange transfusion may be considered [18].

Acquired methemoglobinemia is a frequently overlooked condition that can rapidly compromise a patient's health in hospital settings, particularly with potentially lethal consequences for ICU patients, especially those on ventilators. A notable case report detailed a 46-year-old patient who developed benzocaine-induced methemoglobinemia after an overdose of a 20% benzocaine oral spray (HurriCaine), which was inadvertently left at her bedside by a nurse following a nasogastric procedure [19]. This case highlights the critical need for enhanced awareness and education among healthcare professionals to prevent such errors. Cefalu et al. outlined the diagnostic and therapeutic challenges of methemoglobinemia in the ICU, emphasizing that delays in managing hypoxia or respiratory issues can result in severe outcomes such as cardiac arrest and brain damage [20]. Thus, prompt diagnosis and treatment are essential to prevent severe methemoglobinemia and ensure patient safety.

## Conclusions

The case of benzocaine-induced methemoglobinemia in a patient with multiple comorbidities underscores the importance of exercising caution with this anesthetic drug, particularly in vulnerable populations. Benzocaine use should be avoided in critical care settings for patients with respiratory compromise to prevent potentially severe complications. This incident highlights the critical need for increased awareness among healthcare professionals regarding the risks associated with benzocaine. It is essential to include benzocaine on high-alert medication lists and to improve education on its safe use and the early detection of methemoglobinemia to enhance patient safety.
